# Supplementary material for: A ZKP-based anonymous biometric authentication scheme for the E-health systems
Source: PLoS One. 2025 Jun 17;20(6):e0324289. doi: 10.1371/journal.pone.0324289 (PMC12173353; doi:10.1371/journal.pone.0324289)
Supplement: Attachment — Submitted filename: Response to Reviewers.docx [file pone.0324289.s001.docx]

Dear Editor and Reviewers:

Thank you for your letter and for the valuable feedback provided by both the editor and the reviewers regarding our manuscript titled "A ZKP-Based Anonymous Biometric Authentication Scheme for the E-Health Systems " (ID: PONE-D-24-12359). Those comments are all valuable and helpful for revising and improving our paper, as well as the important guiding significance to our research. We have studied the comments carefully and made corrections, which we hope meet with approval. The revised portions of the manuscript are highlighted in yellow in the paper. The main corrections in the paper and the responds to the reviewer's comments are as follows:

**Responds to the editor's comments:**

First, the study design is not clearly explained. It is unclear how the participants were selected and randomized, and the authors do not provide any information on the blinding of participants or investigators. This could potentially affect the validity of the study results and conclusion should be improved.

Overall, I believe that this manuscript has the potential to make a valuable contribution to the field. However, I recommend that the authors address these concerns before publication as raised during the review process by the reviewers. Thank you for considering my comments. My decision is Minor revision.

Authors are advised to add few more latest citations to improve the quality of research paper.

Chinnasamy, P.; Albakri, A.; Khan, M.; Raja, A.A.; Kiran, A.; Babu, J.C. Smart Contract-Enabled Secure Sharing of Health Data for a Mobile Cloud-Based E-Health System. Appl. Sci. 2023, 13, 3970.

**Response:** Thank you for the suggestion. We have carefully considered your suggestion and have made the necessary changes to the paper. We have added a new subsection titled "Test Databases" within the "Experiments and Evaluations" section. This section details the databases employed for the experiment. Furthermore, in the "Experimental Results" subsection, we have summarized the experimental results, which showed the effectiveness of the ZABA scheme in healthcare environments.

In addition, to enhance the quality of the paper, we have cited this literature in the "Related Work" section, as suggested.

**Responds to the reviewer's comments:**

Reviewer #1:

1. Overall Structure and Clarity

The paper is well-structured, with clear sections and subsections.

However, some sections could benefit from additional explanations or examples to improve clarity, especially for readers who may not be familiar with the technical concepts. Remember to always define acronyms at first use.

**Response:** Thank you for the suggestion. We have revised the manuscript to define all acronyms on their first appearance, as suggested.

2. Acronyms

The acronym "ASS" (Authentication Storage Server) may not be suitable for English readers, as it could be considered inappropriate or offensive. I suggest using a different acronym, such as "AAS" (Authentication and Authorization Server) or "ACS" (Authentication and Credential Server).

Regarding the "AAS" acronym, it seems that the authors intended to use "ASS" (Authentication Storage Server) throughout the paper. However, there is some confusion as they sometimes use "AAS" instead. It would be helpful if the authors clarify and consistently use the same acronym, preferably the suggested "AAS" or "ACS" instead of "ASS."

**Response:** Thank you for suggestion. We have revised the manuscript to replace "ASS" with "AAS" (Authentication and Authorization Server) and reviewed the paper to ensure that the "AAS" is used consistently.

3. Related Work

The related work section provides a comprehensive overview of existing anonymous authentication schemes and biometric authentication techniques.

Suggestion: The authors could consider including a brief discussion on the limitations or gaps in the existing approaches, which their proposed scheme aims to address.

**Response:** Thank you for the suggestion. We have re-written this section according to your suggestion.

4. Proposed Scheme

The proposed ZABA (Zero-Knowledge Proof-Based Anonymous Biometric Authentication) scheme is well-described and supported by theoretical analysis and security proofs.

The authors have clearly outlined the system model, threat model, and the phases involved in the scheme.

The use of multimodal biometrics (fingerprint, face, and iris) is a notable strength of the scheme, as it enhances the security and reliability of the authentication process.

**Response:** Thank you for your positive comment on the proposed ZABA scheme in the paper. We are pleased to hear that our scheme has met your expectations.

5. Experimental Evaluation

The authors have provided detailed information about the implementation and experimental setup, including the hardware configurations and benchmark databases used.

The performance evaluation through experiments and comparisons with existing schemes is thorough and well-presented.

Suggestion: It would be beneficial to include a discussion on the potential limitations or challenges faced during the implementation or experiments, if any.

**Response:** Considering your suggestion, we have added a new paragraph (Paragraph 2) in the "Conclusion" section of the paper. This paragraph discussed the limitations and challenges of the experiment, as well as the possible directions for future work.

6. Here are some additional specific suggestions for improving the clarity and consistency of figures and tables in the paper. In what follows, it is typical that tables and figures should “stand on their own” meaning the reader should be able to gather enough information from the table or figure itself to understand the authors intent.

**Response:** We have carefully considered your suggestion and made the necessary changes to the paper. We have redrawn the tables and figures in the paper by providing detailed captions and thoroughly explained legends.

Special thanks to you for your good comments.

Reviewer #2:

1. The paper needs a proper restructuring to enhance the presentation (for example, the contributions of the paper are presented after related works; this is best presented in the introduction for clarity).

**Response:** Thank you for the suggestion. We have restructured the paper to present the contributions in the "Introduction" section for clarity.

2. To provide more clarity in the abstract, it is important to include more specific details about the methodology and key findings of the proposed scheme (instead of including a statement like "Our approach aims to achieve patient anonymity and authenticity leveraging the Pedersen Vector Commitment algorithm," it will be more appropriate to show the effectiveness of the approach in addressing the aim).

**Response:** Thank you for the suggestion. We have revised the abstract to provide a more detailed and precise overview of the proposed scheme.

3. It is important to discuss the main difference between this paper and the previous version. It is crucial to provide a detailed account of the modifications implemented and the degree to which this paper mirrors the previous version.

**Response:** Thank you for the suggestion. We have added a new paragraph (Paragraph 2) in the "Privacy-preserving Biometric Authentication" subsection of the paper. This paragraph discussed the main difference between this paper and the previous version according to your suggestion.

4. The figure caption should be below the figure and not at the top.

**Response:** We apologize for not placing the figure caption below the figure in the conventional format. We have revised all figures in the manuscript to put the captions below the figures.

5. The theoretical analysis section should focus on evaluation rather than theoretical analysis. This evaluation can also involve testing the approach in a healthcare environment and analyzing the results.

**Response:** Considering your suggestion, we have added a new subsection entitled "Test Databases" in the "Experiments and Evaluations" section of our paper. Furthermore, we have expanded the "Experimental Results" subsection to include testing and analysis of our ZABA scheme within a healthcare environment.

6. I recommend citing more relevant literature to enhance the paper's theoretical background.

**Response:** Thank you for the suggestion. We have re-written the "Related Work" section and cited more relevant literature to enhance the paper's theoretical background.

7. I also recommend language editing for the paper to improve its presentation.

**Response:** Thank you for the suggestion. We have polished the paper to ensure the content is explicit, grammatically correct, and well-structured.

Special thanks to you for your good comments.

We appreciate for Editors/Reviewers' warm work earnestly and hope that the correction will meet with approval.

Once again, thank you very much for your comments and suggestions.
