# Supplementary material for: A ZKP-based anonymous biometric authentication scheme for the E-health systems
Source: PLoS One. 2025 Jun 17;20(6):e0324289. doi: 10.1371/journal.pone.0324289 (PMC12173353; doi:10.1371/journal.pone.0324289)
Supplement: Attachment — Submitted filename: Comment for PLOS manuscript.docx [file pone.0324289.s002.docx]

**Comments**

Page 1, Lines 8-15 Comment: The abstract should provide specific details about the methodology, such as key metrics (e.g., accuracy, computational cost) and evaluation outcomes. Replace vague phrases like "the effectiveness and practicality of our scheme" with concrete results.

Inconsistent Acronym Usage:

Page 1, Line 20, and throughout the manuscript Comment: Ensure consistent use of the acronym "AAS" for "Authentication and Authorization Server" throughout the manuscript. Double-check for any remaining instances of "ASS."

Introduction Depth: Page 2, Lines 10-15 Comment: Expand on how the proposed ZABA scheme improves upon prior ZKP-based solutions, particularly in terms of efficiency, security, and practical deployment in e-health systems.

Page 3, Lines 5-10 Comment: Add a summary that explicitly identifies gaps in the literature and connects them directly to the proposed contributions of this study.

Consistency in Figure Captions: Figures (Pages 9 and 12) Comment: Ensure all figure captions are placed below the figures and provide self-explanatory details. For example, the "Simple Diagram of the ZABA scheme" caption should briefly explain its significance in the authentication process.

Justification of Methodology: Page 8, Lines 15-20 Comment: Provide a clearer justification for choosing the Pedersen vector commitment algorithm over other cryptographic methods. Discuss its specific advantages in the context of e-health.

Evaluation Metrics Explanation: Page 14, Line 5 Comment: Explain why Euclidean distance is chosen as the primary metric for identity authentication and how the threshold (≤4) was determined.

Grammar and Syntax Corrections: Throughout the manuscript (e.g., Page 2, Line 5) Comment: Revise grammar and syntax issues, such as "The e-health system oers" to "The e-health system offers." Perform a thorough language edit to improve readability.

Highlight Practical Challenges: Page 15, Lines 10-15 Comment: Discuss the challenges faced during experimental implementation, such as computational overhead or real-time data acquisition limitations, and their potential resolutions.

Conclusion Enhancement: Page 16, Lines 5-10 Comment: Expand the conclusion by briefly discussing future enhancements, such as testing with larger datasets or optimizing the scheme for low-power devices.
