# Supplementary material for: A ZKP-based anonymous biometric authentication scheme for the E-health systems
Source: PLoS One. 2025 Jun 17;20(6):e0324289. doi: 10.1371/journal.pone.0324289 (PMC12173353; doi:10.1371/journal.pone.0324289)
Supplement: Attachment — Submitted filename: Response_to_Reviewers_auresp_1.docx [file pone.0324289.s004.docx]

Dear Editor and Reviewers:

Thank you for your letter and for the valuable feedback provided by both the editor and the reviewers regarding our manuscript titled "**A ZKP-Based Anonymous Biometric Authentication Scheme for the E-Health Systems " (ID: PONE-D-24-56772)**. Those comments are all valuable and helpful for revising and improving our paper, as well as the important guiding significance to our research. We have studied the comments carefully and made corrections, which we hope meet with approval. The revised portions of the manuscript are highlighted in yellow in the paper. The main corrections in the paper and the responds to the reviewer's comments are as follows:

**Responds to the reviewer's comments:**

**Reviewer #1:**

Please add the contribution of all the authors and acknowledge the funding agency. The authors have written the manuscript in a well-mannered manner with substantial data and discussion. The data of the current manuscript is enough for publication in journal like PLOS one.

**Response:** Thank you for your positive feedback and suggestion. We have included the contribution details of all authors and acknowledged the funding agency in the Cover Letter. We appreciate your recognition of our work and the quality of our data and discussion.

1. Page 1, Lines 8-15 Comment: The abstract should provide specific details about the methodology, such as key metrics (e.g., accuracy, computational cost) and evaluation outcomes. Replace vague phrases like "the effectiveness and practicality of our scheme" with concrete results.

**Response:** Thank you for your suggestion. We have revised the abstract by clearly specifying the key metrics to present concrete results. To enhance clarity and precision, we have replaced vague expressions with explicit descriptions that better reflect our scheme.

2. Page 1, Line 20, and throughout the manuscript Comment: Ensure consistent use of the acronym "AAS" for "Authentication and Authorization Server" throughout the manuscript. Double-check for any remaining instances of "ASS."

**Response:** Thank you for your suggestion. We have thoroughly reviewed and revised the manuscript to ensure the consistent use of the acronym "AAS" for "Authentication and Authorization Server."

3. Page 2, Lines 10-15 Comment: Expand on how the proposed ZABA scheme improves upon prior ZKP-based solutions, particularly in terms of efficiency, security, and practical deployment in e-health systems.

**Response:** Thank you for your suggestion. As your recommendation, we have highlighted how the proposed ZABA scheme improves upon previous ZKP-based solutions in the contributions section.

4. Page 3, Lines 5-10 Comment: Add a summary that explicitly identifies gaps in the literature and connects them directly to the proposed contributions of this study.

**Response:** Thank you for your suggestion. We have revised the Related Work section to explicitly identify gaps in the literature and establish a direct connection between these gaps and the contributions of our study.

5. Figures (Pages 9 and 12) Comment: Ensure all figure captions are placed below the figures and provide self-explanatory details. For example, the "Simple Diagram of the ZABA scheme" caption should briefly explain its significance in the authentication process.

**Response:** Thank you for your suggestion. We have carefully revised the figure captions to ensure they are correctly positioned below the corresponding figures. Additionally, we have provided self-explanatory details for all figures.

6. Page 8, Lines 15-20 Comment: Provide a clearer justification for choosing the Pedersen vector commitment algorithm over other cryptographic methods. Discuss its specific advantages in the context of e-health.

**Response:** Thank you for your suggestion. In the Assumption and Commitment section, we provide a reason for selecting the Pedersen vector commitment algorithm.

7. Page 14, Line 5 Comment: Explain why Euclidean distance is chosen as the primary metric for identity authentication and how the threshold (≤4) was determined.

**Response:** Thank you for your suggestion. We have added an explanation in the Implementation section, clarifying the rationale behind our choice.

8. Throughout the manuscript (e.g., Page 2, Line 5) Comment: Revise grammar and syntax issues, such as "The e-health system oers" to "The e-health system offers." Perform a thorough language edit to improve readability.

**Response:** Thank you for your suggestion. We have carefully revised the manuscript to correct grammar and syntax issues.

9. Page 15, Lines 10-15 Comment: Discuss the challenges faced during experimental implementation, such as computational overhead or real-time data acquisition limitations, and their potential resolutions.

**Response:** Thank you for your suggestion. We have expanded the discussion in the Test Databases section to address the challenges encountered during experimental implementation.

10. Page 16, Lines 5-10 Comment: Expand the conclusion by briefly discussing future enhancements, such as testing with larger datasets or optimizing the scheme for low-power devices.

**Response:** Thank you for your suggestion. In response, we have expanded the conclusion to discuss potential future enhancements.

Special thanks to you for your good comments.

**Reviewer #2:**

The manuscript titled ‘A ZKP-Based Anonymous Biometric Authentication Scheme for E-Health Systems’ is well-structured and presents an interesting approach. However, several aspects require significant improvement. I suggest revisions that will enhance its clarity and impact.

1. The abstract should be revised to remove redundant descriptions of the benefits of e-health systems, like improved accessibility, efficiency, and patient experience and the security aspects of the proposed scheme. Additionally, the phrase ‘and more’ is vague and unnecessary and should be omitted for clarity.

**Response:** Thank you for your suggestion. We have revised the abstract to remove redundant descriptions and vague terms, ensuring clarity.

2. I suggest highlighting specific contributions or key insights gained from this study in the abstract. This will help differentiate your study from existing literature reviews on the topic and emphasize its novelty.

**Response:** Thank you for your suggestion. We have revised the abstract to highlight the key contributions and novel insights of our study.

3. Define each acronym like ‘IoT’ at its first use. Check through the entire manuscript to make sure it is defined at the first use.

**Response:** Thank you for your suggestion. We have carefully reviewed the manuscript and ensured that each acronym is defined upon its first use for clarity and consistency.

4. The manuscript discusses multimodal biometrics and cancelable biometrics (MCBG) for security, given that multimodal biometrics can still be vulnerable to presentation attacks. However, it does not mention any anti-spoofing techniques. How does your scheme handle biometric spoofing attacks?

**Response:** Thank you for your suggestion. We have included a detailed explanation in the Multimodal Cancelable Biometric Technique section. Specifically, we emphasize the use of irreversible transformations, key image revocation, and adaptive processing techniques to enhance resilience against presentation attacks.

5. The theoretical analysis section should focus on evaluation rather than theoretical analysis. This evaluation can also involve testing the approach in a healthcare environment and analyzing the results.

**Response:** Thank you for your suggestion. We have added a detailed performance evaluation in the Experimental Evaluation and Results section. Specifically, we have incorporated an assessment of our scheme in a healthcare environment using the MIMIC-III dataset.

6. The English of the manuscript needs to be improved.

**Response:** Thank you for the suggestion. We have carefully revised the manuscript to enhance clarity, refine grammar, and improve overall readability.

7. The conclusion section should be revised and concise.

**Response:** Thank you for your suggestion. We have revised the conclusion section to enhance clarity and conciseness.

Special thanks to you for your good comments.

We appreciate for Editors/Reviewers' warm work earnestly and hope that the correction will meet with approval.

Once again, thank you very much for your comments and suggestions.
